# An Automatic Exposure Method of Plane Array Remote Sensing Image Based on Two-Dimensional Entropy

**DOI:** 10.3390/s21103306

**Published:** 2021-05-11

**Authors:** Tan Gao, Liangliang Zheng, Wei Xu, Yongjie Piao, Rupeng Feng, Xiaolong Chen, Tichao Zhou

**Affiliations:** 1 Changchun Institute of Optics, Fine Mechanics and Physics, Chinese Academy of Sciences, Changchun 130033, China; gaotan19@mails.ucas.ac.cn (T.G.); zhengliangliang@ciomp.ac.cn (L.Z.); pyj0314@163.com (Y.P.); fengrupeng@ciomp.ac.cn (R.F.); chenxiaolong20@mails.ucas.ac.cn (X.C.); zhoutichao@ciomp.ac.cn (T.Z.); 2 University of Chinese Academy of Sciences, Beijing 100039, China; 3 Key Laboratory of Space-Based Dynamic & Rapid Optical Imaging Technology, Chinese Academy of Sciences, Changchun 130033, China

**Keywords:** exposure time, two-dimensional entropy, threshold, cubic spline, image details

## Abstract

The improper setting of exposure time for the space camera will cause serious image quality degradation (overexposure or underexposure) in the imaging process. In order to solve the problem of insufficient utilization of the camera’s dynamic range to obtain high-quality original images, an automatic exposure method for plane array remote sensing images based on two-dimensional entropy is proposed. First, a two-dimensional entropy-based image exposure quality evaluation model is proposed. The two-dimensional entropy matrix of the image is partitioned to distinguish the saturated areas (region of overexposure and underexposure) and the unsaturated areas (region of propitious exposure) from the original image. The ratio of the saturated area is used as an evaluating indicator of image exposure quality, which is more sensitive to the brightness, edges, information volume, and signal-to-noise ratio of the image. Then, the cubic spline interpolation method is applied to fit the exposure quality curve to efficiently improve the camera’s exposure accuracy. A series of experiments have been carried out for different targets in different environments using the existing imaging system to verify the superiority and robustness of the proposed method. Compared with the conventional automatic exposure method, the signal-to-noise ratio of the image obtained by the proposed algorithm is increased by at least 1.6730 dB, and the number of saturated pixels is reduced to at least 2.568%. The method is significant to improve the on-orbit autonomous operating capability and on-orbit application efficiency of space camera.

## 1. Introduction

With the rapid development of space remote sensing technology, the strong demand for satellite responsiveness and imaging quality are increasing [1]. The imaging parameters of space camera are usually determined by the satellite earth station according to factors such as the solar altitude angle α, the ground object reflectivity ρ, and the weather of the target location before a conventional space camera system performs a shot task. When the satellite passes through the border, it is uploaded to the satellite system through an available satellite earth station. When the satellite reaches the target location, the imaging parameters are used to set the operative condition of the space camera to detect and collect the terrain object information. The a priori model is based on ideal conditions and often cannot get ideal results in the actual imaging process.

Space cameras are different from ubiquitous cameras. It has a high dynamic range (HDR) and large area of the shooting scene, and the obtained remote sensing image has a large amount of data and rich details which all lead to higher exposure quality of the space camera [2,3]. The current conventional camera automatic exposure method can be divided into three categories: the determination of imaging parameters based on the prior model, image fusion and the determination of imaging parameters based on image statistics.

The first method is to use a priori knowledge based on the scene to set imaging parameters. Wang et al. proposed a prediction method of optimal parameters combination, i.e., inputting empirical target refraction and image digital number (DN) to radiative transfer model to output the target radiance expected, thus obtaining an expected absolute radiometric coefficient before launch, which could be helpful for rational use of relevant earth observation cameras [4]. Cao et al. proposed a method for autonomous imaging parameters adjustment based on solar elevation from remote sensing theory [5]. The factors which influence imaging quality and the relationship between apparent radiance and solar elevation are firstly discussed. Then, the integral time change under different roll angles in one orbital period and the internal links among solar elevation, roll angle, integral grade, and gain are analyzed. Finally, the best grading strategy is obtained and the two-dimensional lookup table which can be used for the autonomous imaging parameters adjustment is built. The strain capacity of the method is poor. If the weather conditions of the target imaging area change, and the camera system still looks up the table according to the original parameter state, the acquired image data may not achieve the anticipant imaging effect, and even the imaging task is invalid. The main disadvantage is that the camera system cannot adjust the imaging parameters adaptively based on the actual image data acquired.

The method of image fusion does not need to adjust the camera imaging parameters, but only needs to acquire multiple images. Traditional exposure fusion methods can generate HDR images based on a set of weight maps of low dynamic range (LDR) images with different exposures [6]. There are a variety of image fusion algorithms, from simple weighted average to complex methods based on advanced statistical image models. Ying et al. first used the illumination estimating technology to calculate the weight matrix for the image, and then used the camera’s response model to synthesize the multiple exposure image to get the optimal exposure rate [7]. This method performs well in the underexposed areas, but performs badly in the overexposed areas of the original image. Mertens et al. used three quality indices of brightness, contrast, and saturation to determine the effect of a given pixel on the final composite image. The fusion image has a high contrast, but still cannot display the details of the brightest and darkest areas of the scene [8]. Effective image fusion methods require multiple pre-acquired images of different dynamic range [9], for the shooting target under extreme lighting conditions is poor.

Another method is to establish the mathematical relationships between image quality indices and imaging parameters through the analysis of image statistics, thereby adjusting the camera’s imaging parameters such as shutter speed, aperture size, exposure time, and gain. The adjustment of shutter speed and aperture size usually depends on a high-precision mechanical structure, and the adjustable imaging parameters of the plane array space camera are usually exposure time and gain.

The simplest method is to measure the average gray value of the entire image or a specific area, and adjust the imaging parameters of the camera to make the average gray value of the image equal to half of the camera’s dynamic range [10,11]. Kuno et al. calculated the average brightness of the entire image when analyzing the brightness of the image, and used the average brightness to indicate the brightness level of the entire image [12]. Then, set a target brightness and adjust the imaging parameters to make the brightness level of the image gradually consistent with the target brightness. This method based on the average gray value of the image could cause a large area of overexposure and underexposure at the same time, resulting in the loss of a large amount of image details.

Later scholars used more advanced image quality evaluation indices such as image gray histogram, one-dimensional entropy, and gradient to obtain the optimal exposure image. Montalvo et al. extracted the histogram of the region of interest from the histogram of the R and G channels in the RGB spectral channel, and then used the brightness of this area as the reference brightness to adjust the imaging parameters of the camera by the histogram matching method [13]. Torres et al. shifted the grayscale histogram of the image to a specified range by adjusting the imaging parameters of the camera, which can avoid overexposure or underexposure of the image to a certain extent [14]. Rahman et al. and Lu et al. proposed an automatic exposure method that uses the maximum image entropy as the image quality evaluation index [15,16]. Research shows that the size of entropy changes with the exposure parameters of the imaging system, and the imaging parameter corresponding to the largest entropy value is taken as the optimal imaging parameter. Zhang et al. proposed an active exposure control method to maximize the gradient information in the image [17]. They calculated the derivative of the gradient square and the photometric response function, and measured the change of the gradient with the exposure time to determine the optimal exposure time. Shim et al. also used the gradient information in the image to get the appropriate exposure time [18,19]. The author defines an information metric based on the size of the gradient at each pixel, and simulates exposure changes by applying different gamma corrections to the original image to find the gamma value that maximizes the gradient information, and then adjust the exposure time according to the gamma value. The above algorithms cannot behave well when both image details and brightness are concerned.

Recently, Kim et al. proposed a new exposure quality evaluation method, of which the entropy weighted gradient of the image was used as an image quality evaluation index [20,21]. They used the index to obtain the optimal exposure time of the camera. The entropy matrix partitioning threshold is a fixed value in this method because of the small shooting area with a large target, and it has a better shooting effect on scenes with less detail. Remote sensing images are rich in details, and the target is usually only a few pixels in size which can lead to massive loss in image details. Each of these methods has advantages and disadvantages.

Aiming at these problems above, some research work on automatic exposure methods for plane array remote sensing images is carried out in this paper. A new image exposure quality evaluation model for remote sensing and an optimal exposure time determination method are proposed. We conducted experiments under different conditions to verify the robustness of the method. Experimental results show that, compared with the current algorithm, the algorithm is more sensitive to image details, brightness, information, and signal-to-noise ratio, which perfectly meets the quality requirements of remote sensing images.

The rest of the paper is organized as follows: the automatic exposure method for plane array remote sensing images based on two-dimensional entropy is proposed in Section 2; in Section 3, the proposed algorithm is experimentally compared with other algorithms and related discussions are carried out; Section 4 presents the conclusions of the paper.

## 2. Proposed Algorithm

The adjustable imaging parameters of the area array space camera are usually exposure time and gain. The relationship between the exposure quality and the exposure time of the space camera is mainly studied in this paper, so as to get the optimal exposure time in the imaging process. Similar to the previous research [20,21], considering the sensitivity of two-dimensional entropy to image brightness, edges, and information, the two-dimensional entropy of the image is used as the starting point. Different from previous studies, an adaptive two-dimensional entropy matrix partitioning threshold based on the maximum weighted variance is proposed, which can be used to distinguish the saturated and unsaturated regions of the image. Then, the cubic spline interpolation method is introduced to calculate the optimal exposure time. The algorithm proposed in this paper takes the proportion of the saturated area in the image as a measure. With good sensitivity to image brightness, edges, information, and signal-to-noise ratio, it can also maximize the reduction of overexposed and underexposed areas in the image. The proposed algorithm has a good adaptability to remote sensing imaging and important guiding significance to obtain high-quality original remote sensing images for the space camera.

**Figure 1:**
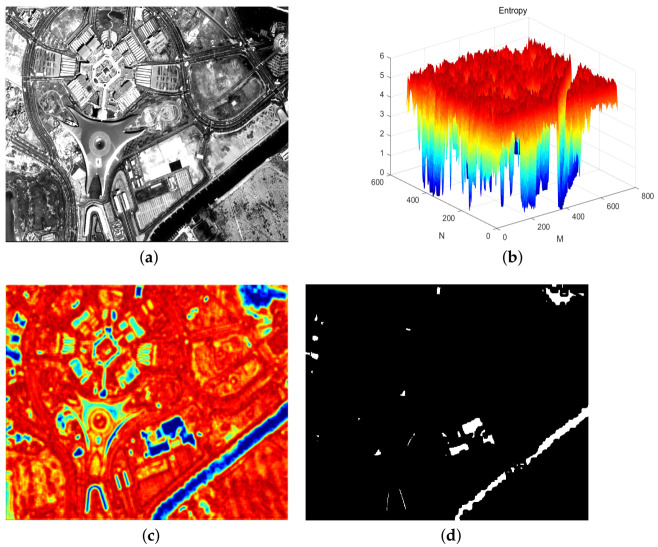
(**a**) Original image; (**b**) three-dimensional view of entropy matrix; (**c**) top view of entropy matrix; (**d**) segmentation of saturated regions.

It can be clearly seen from Figure 1 that the entropy value of the saturated area of the image is small, while the entropy value of the unsaturated area is larger. The image segmentation as shown in Figure 1d can be achieved by getting an appropriate entropy threshold.

**Figure 2:**
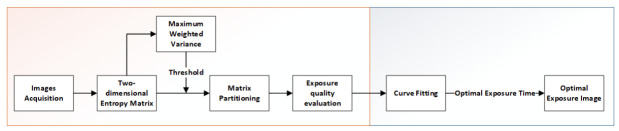
Overall architecture of the proposed algorithm.

The overall architecture of the algorithm in this paper is shown in Figure 2. The method consists of two modules: exposure quality evaluation module and exposure curve fitting module. First, λ is supposed as the exposure time step to acquire multiple images; then, the exposure quality of the collected images is calculated; then, we perform curve fitting on the exposure quality of these images; finally, the optimal exposure time is acquired according to the fitted curve.

### 2.1. Two-Dimensional Entropy of Image

The one-dimensional entropy of image, as the information feature of the image, cannot map the spatial distribution of pixels well. The advantage of using two-dimensional entropy as an image saturation measure is that the two-dimensional entropy of an image is more sensitive to features such as brightness, edges, and information. For remote sensing images, there are certain grayscale changes in the target areas, while the grayscales of pixels in the overexposed and underexposed areas are keeping consistent basically which provides a good prerequisite for using two-dimensional entropy as a saturation measure.

The definition of Shannon entropy is used in this paper [22], the two-dimensional entropy of the pixel $I_{i,j}$ in the image is:

(1) $$H_{i,j}=-\sum P_{GS_{i,j}}\log_{10}P_{GS_{i,j}}$$

where $GS_{i,j}$ indicates the gray level corresponding to pixel $I_{i,j}$, $P_{GS_{i,j}}$ indicates the probability that the corresponding gray level of the pixel $I_{i,j}$ in the 9×9 neighborhood.

### 2.2. Entropy Matrix Partitioning Threshold

The original image can be divided into region of saturation (ROS) and region of unsaturation (ROUS) by choosing an appropriate threshold to partition the two-dimensional entropy matrix because of the above-mentioned characteristics of the two-dimensional entropy matrix. The following method is adopted to obtain the partitioning threshold of the two-dimensional entropy matrix.

Assuming the threshold is th, when $H_{i,j}<th$, it is defined as the ROS entropy matrix element $H_{ROS_{i,j}}$. On the contrary, when $H_{i,j}>th$, it is defined as the ROUS entropy matrix element $H_{ROUS_{i,j}}$. The variances of the two-dimensional entropy matrix of the saturated and unsaturated regions are calculated respectively:

(2) $$\sigma_{ROS}^{2}=\sum {\left(H_{ROS_{i,j}}-mean_{H_{ROS}}\right)}^{2}$$

(3) $$\sigma_{ROUS}^{2}=\sum {\left(H_{ROUS_{i,j}}-mean_{H_{ROUS}}\right)}^{2}$$

where $\sigma_{ROS}^{2}$ and $\sigma_{ROUS}^{2}$ respectively indicate the variance of the entropy matrix of the saturated region and the unsaturated region, $mean_{H_{ROS}}$ and $mean_{H_{ROUS}}$ respectively indicate the mean value of the entropy matrix elements of the saturated region and the unsaturated region, $H_{ROS_{i,j}}$ and $H_{ROUS_{i,j}}$ respectively represent the elements of the saturated and unsaturated regions in matrix *H*.

Derived from (2) and (3), the total weighted variance of the two-dimensional entropy matrix *H* is expressed as:

(4) $$\sigma_{H}^{2}=p_{ROS}\;\sigma_{ROS}^{2}+p_{ROUS}\;\sigma_{ROUS}^{2}$$

where $p_{ROS}$ and $p_{ROUS}$ respectively indicate the proportion of ROS element and the proportion of ROUS element in the whole image when the threshold is th. Substituting (2) and (3) into (4):

(5) $$\sigma_{H}^{2}=p_{ROS}\sum {\left(H_{ROS_{i,j}}-mean_{H_{ROS}}\right)}^{2}+p_{ROUS}\sum {\left(H_{RONS_{i,j}}-mean_{H_{ROUS}}\right)}^{2}$$

For (5), there is a matrix as follows:

(6) $$th_{k}={\left[minH_{i,j},minH_{i,j}+0.0001,minH_{i,j}+0.0002,\cdots ,maxH_{i,j}\right]}_{1\times n}$$

Traverse each element in $th_{k}$ and calculate all corresponding $\sigma_{H}^{2}$. When $\sigma_{H}^{2}$ gets the maximum value, the corresponding $th_{k}$ is the optimal threshold, which is expressed as:

(7) $$\mathrm{th}=\arg\;max\left[\sigma_{H}^{2}\left(th_{k}\right)\right]$$

in the equation, $max\left[\sigma_{H}^{2}\left(th_{k}\right)\right]$ indicates the maximum value of $\sigma_{H}^{2}$.

### 2.3. Ratio of Saturated Area

Based on the results obtained in Section 2.2, a binary mask is defined to partition the two-dimensional entropy matrix and distinguish ROS and ROUS:

(8) $${\mathrm{Mask}}_{i,j}=\left\{\begin{array}{c} 0,\;th\leq H_{i,j}\\ 1,\;0\leq H_{i,j}<th \end{array}\right.$$

According to the defined mask matrix $Mask_{i,j}$, the value of the corresponding element of the ROS area is 1 and the value of the corresponding element of ROUS is 0. The proportion of ROS elements in the image can be obtained as:

(9) $$S=\sum_{i,j}^{m,n}Mask_{i,j}/\left(m\times n\right)$$

where *m* and *n* indicate the image size. Obviously, when the *S* value in (9) is smaller, the image ROS ratio is lower and the image exposure quality is better; when the *S* value is larger, the image ROS ratio is higher and the image exposure quality is worse. In the latter part of this paper, the *S* value is used as the image exposure quality evaluation standard, which is used as the basis for adjusting the camera’s exposure time.

### 2.4. Exposure Quality Curve Fitting

For a certain scene, the plane array space camera can continuously expose the specified target when it is in the staring imaging mode. When shooting with an equal step of exposure time, the corresponding *S* value can be calculated for each image. We use the exposure time as the abscissa and the *S* value as the ordinate for curve fitting. Choosing a suitable curve fitting algorithm is the key to accurately determine the optimal exposure time. In this paper, the cubic spline interpolation method is used to fit the discrete points into a curve to show the relationship between the image exposure quality and the exposure time, so that the exposure time can be adjusted more accurately and the image with higher exposure quality can be obtained.

Cubic spline interpolation fits a smooth curve from a series of sample points. Mathematically, it is the process of obtaining a set of curve functions by solving the three-moment equation. Due to the small computation capacity and low complexity, cubic spline interpolation is widely used in various fields. The cubic spline curve about exposure time and image exposure quality can be expressed as:

(10) $$S_{i}\left(t\right)\;=a_{i}{\left(t-t_{i}\right)}^{3}+b_{i}{\left(t-t_{i}\right)}^{2}+c_{i}\left(t-t_{i}\right)\;+\;d_{i}\;\;,\;\;i=1,2,3,\cdots n-1,n$$

in the (10), *t* is the exposure time, and $t_{i}$ is the abscissa of the sample point. $a_{i}$, $b_{i}$, $c_{i}$ and $d_{i}$ are undetermined coefficients. $S_{i}\left(t\right)$ is the exposure quality corresponding to the exposure time *t*. Different from the conventional cubic spline interpolation method, the boundary condition of the spline curve in this paper is the Not-A-Knot [23]:

(11) $$\left\{\begin{array}{c} S_{0}^{{}^{\prime \prime \prime }}\left(t_{0}\right)\;=S_{1}^{{}^{\prime \prime \prime }}\left(t_{1}\right)\\ S_{n-1}^{{}^{\prime \prime \prime }}\left(t_{n-1}\right)\;=S_{n}^{{}^{\prime \prime \prime }}\left(t_{n}\right) \end{array}\right.$$

where $S_{n}^{{}^{\prime \prime \prime }}\left(t_{n}\right)$ indicates the third derivative of the curve at point $t_{n}$.

Fitting the exposure quality curve based on cubic spline interpolation Not-A-Knot boundary conditions has the following advantages:

(1) Interpolation can still be performed when there are few sample points;
(2) There will be no excessive errors at the start and end points of the sample;
(3) It can characterize the change of exposure quality with exposure time at different exposure time steps;
(4) The second-order smoothness of the curve conforms to the gradual characteristic of the exposure quality with the exposure time.

### 2.5. Rules for Determining the Optimal Exposure Time

Generally, the exposure quality curve of the images is a concave curve. The optimal exposure image corresponds to the lowest point of the curve from the above. When the accuracy of the target exposure time is $\lambda_{\min}$, suppose that the exposure time $\lambda =2\lambda_{\min}$ for continuous exposure to obtain multiple sample points. According to the fitted curve, the optimal exposure time can be extracted. For different shooting scenes, the relationship between image exposure quality and exposure time does not strictly satisfy the cubic spline function. Therefore, the following optimal exposure time determining rules are proposed to avoid the errors:

(12) $$t_{optimal}=\;\left\{\begin{array}{c} \arg\;min\left[S\left(t\right)\right],\;\;min\left[S\left(t\right)\right]\;=min\left[SP\left(t\right)\right]\\ \arg\;min\left[S_{a}\left(t\right),min\left[SP\left(t\right)\right]\right],\;\;min\left[S\left(t\right)\right]\;\neq min\left[SP\left(t\right)\right]\; \end{array}\right.$$

where $t_{optimal}$ indicates the optimal exposure time of the scene. $minS\left(t\right)$ indicates the minimum value obtained by sampling in the curve with $\lambda_{\min}$ as the step size. $minSP\left(t\right)\;$ indicates the minimum value of the sample point, and $S_{a}\left(t\right)$ indicates the *S* value of the actual image obtained by configuring the camera parameters with the exposure time corresponding to the lowest point of the curve.

## 3. Verification and Analysis

In order to verify the algorithm proposed, we conducted multiple sets of experiments to simulate low ground reflectivity and high ground reflectivity in different scenes. First, the image exposure quality evaluation model proposed in this paper is verified; then, the performance of the cubic spline interpolation curve in improving the exposure accuracy of the imaging system and improving the image exposure quality is verified. In the experiment, the camera’s default method (CDM), average gray level method (AGLM) [12], one-dimensional entropy method (ODEM) [15], entropy weighted gradient method (EWGM) [21], gradient-based method (GBM) [19], etc. are used for comparison, which proves the superiority of the proposed method.

The camera used in the experiment is acA1300-60gm from Basler. The parameter indices are shown in Table 1.

In the experiment, the relative aperture value of the camera is 1:1.4 and the image size is 1280×1024.

### 3.1. Experiment under Bright Conditions

**Figure 3:**
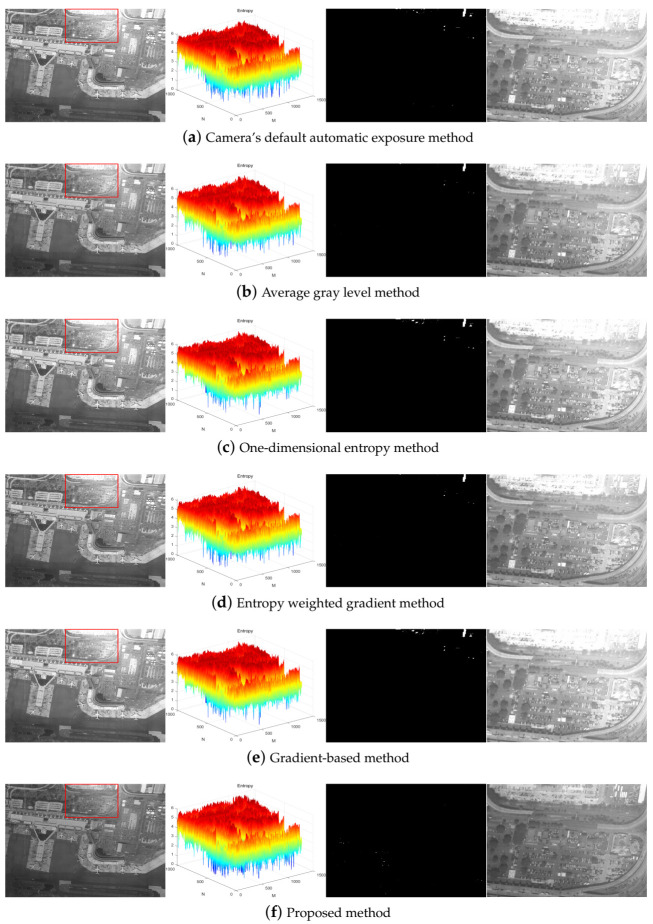
The optimal exposure image, the three-dimensional image entropy matrix, segmentation of ROS, and the enlarged view of the labeled region of each method under bright conditions from left to right.

Suppose that the exposure time step $\lambda_{1}=20$ ms under bright conditions to take eight consecutive shots of target 1. The exposure quality is the highest when the exposure time is 41 ms. The optimal exposure image obtained by other algorithms is also shown in Figure 3.

Figure 3 shows that the default automatic exposure algorithm of the camera is similar to the average gray level method, which has certain advantages in the overall brightness of the image, but the overexposed area in the image is not effectively limited. The one-dimensional entropy method pays attention to the overall information of the image. The result of the gradient-based method is consistent with the one-dimensional entropy method, the contrast of the image is relatively strong, but the overexposure phenomenon also exists. The entropy weighted gradient method comprehensively considers the amount of information and edge details of the image, which makes overexposure and underexposure of the image suppressed to a certain extent, but information loss in some areas is still unavoidable. The optimal exposure image obtained by the proposed algorithm has a weaker contrast, but the blue part of the entropy matrix is the least. In other words, the overexposure and underexposure areas in the entire image are minimized so that the image details are preserved to the maximum extent.

Some other objective indices were used to compare the optimal exposure images of each method, as shown in Table 2. Table 2 shows that the optimal exposure image taken by the proposed algorithm has a small gradient, but the number of saturated pixels is much lower than that of other algorithms, and the signal-to-noise ratio is increased by at least 2.4031 dB.

The average gradient equation in this paper is expressed as:

(13) $$G=\frac{1}{m\times n}\sum_{i=1}^{m}\sum_{j=1}^{n}\sqrt{\frac{{\left(\frac{\partial f}{\partial x}\right)}^{2}+{\left(\frac{\partial f}{\partial y}\right)}^{2}}{2}}$$

where $\frac{\partial f}{\partial x}$ indicates the gradient in the horizontal direction and $\frac{\partial f}{\partial y}$ indicates the gradient in the vertical direction, *m* and *n* indicate the image size.

SNR formula is expressed as:

(14) $$\mathrm{SNR}=20\mathrm{lo}g_{10}\frac{I_{max}-I_{min}}{\sigma }$$

where $I_{max}$ and $I_{min}$ respectively indicate the maximum and minimum values of the image gray level, and σ represents the standard deviation of the image.

**Figure 4:**
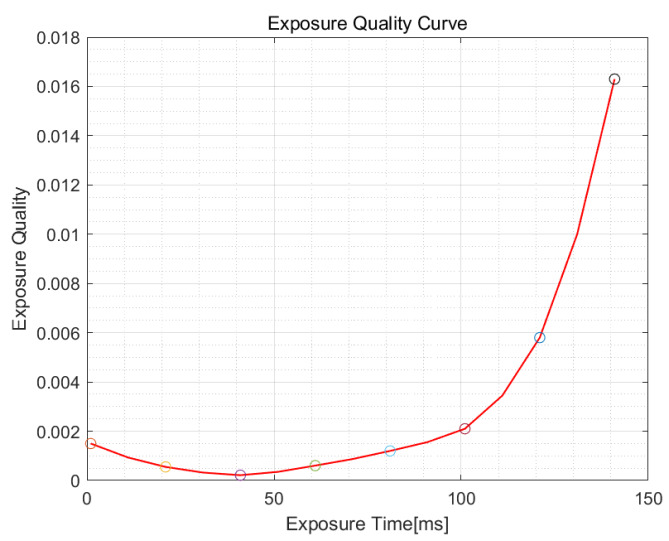
Curve of exposure quality under bright conditions.

In order to further improve the exposure accuracy and the exposure quality, curve fitting is performed on the *S* value of the obtained images. Sampling with ${\lambda_{1}}^{{}^{\prime }}$ = 10 ms in the curve. The resulting curve is shown in Figure 4.

It can be seen from the curve that the optimal exposure time is 41 ms. Through actual shooting, the quality of the image taken at exposure timing of 41 ms is better than that at exposure timing of 31 ms and 51 ms. The determination of the optimal exposure time is accurate, which verifies the accuracy of the algorithm proposed.

### 3.2. Experiment under Dark Conditions

**Figure 5:**
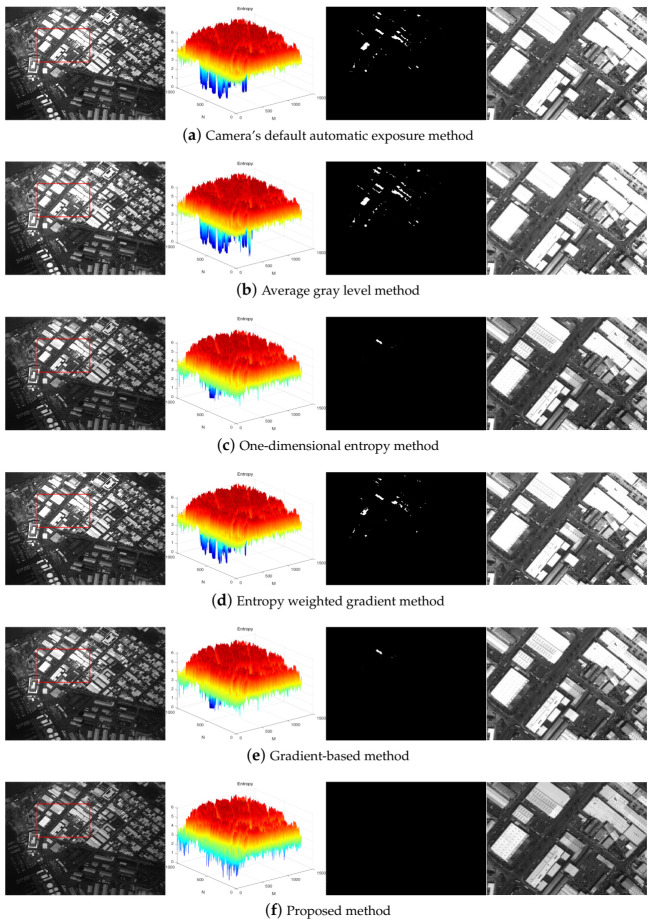
The optimal exposure image, the three-dimensional image entropy matrix, segmentation of ROS, and the enlarged view of the labeled region of each method under dark conditions from left to right.

Suppose that the exposure time step $\lambda_{2}$ = 50 ms under dark conditions to take eight consecutive shots of target 2. The exposure quality is the highest when the exposure time is 205 ms. The optimal exposure image obtained by other algorithms is shown in Figure 5.

Figure 5 shows that the experimental results are consistent with the experimental results under bright conditions, and more intuitively reflecting the superiority of the algorithm. Figure 5 shows that the experimental results are consistent with the experimental results under bright conditions, and more intuitively reflecting the superiority of the algorithm. The camera’s default automatic exposure algorithm is similar to the average gray scale method. It has certain advantages in the overall brightness of the image, but the overexposed area in the image is not limited. The one-dimensional entropy method pays attention to the overall information of the image. The result of the gradient-based method is consistent with the one-dimensional entropy method. The contrast of the image is relatively strong, but it also cannot limit the overexposure phenomenon. The entropy weighted gradient method comprehensively considers the amount of information and edge details of the image, which makes overexposure and underexposure of the image suppressed to a certain extent, but information loss in some areas is still unavoidable. The entropy matrix of the optimal exposure image obtained by the proposed algorithm has the least blue part and the smallest saturated area of the image, which can clearly show the detailed information of the marked area and even the whole image.

Some other objective indices were used to compare the optimal exposure images obtained by each method. As shown in Table 3, the optimal exposure image taken by the proposed algorithm in this scene has the smallest average gradient, but the number of saturated pixels is much lower than that of other algorithms. The signal-to-noise ratio is improved by at least 1.6730 dB.

According to the curve, the optimal exposure time is 180 ms. However, the actual quality of the image taken at exposure timing of 180 ms is worse than that at exposure timing of 205 ms. According to the rules for determining the optimal exposure time defined in Section 2.5, the optimal exposure time in this scene is 205 ms. It verifies the necessity of the rules in Section 2.5.

**Figure 6:**
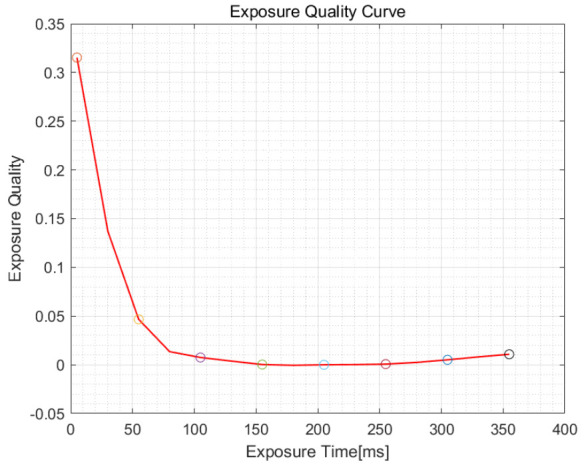
Curve of exposure quality under dark conditions.

In order to further improve the exposure accuracy and improve the exposure quality, we fit the S value of the images obtained by the proposed algorithm to a curve, and sample it with ${\lambda_{2}}^{{}^{\prime }}$ = 25 ms. The resulting curve is shown in Figure 6.

### 3.3. Experiment Results Analysis

The above experimental results show that there are partial overexposure and underexposure in the images taken by the conventional automatic exposure algorithm, resulting in the loss of information of the image. The algorithm proposed can effectively solve this problem. Compared with several other algorithms, the algorithm proposed has better subjective and objective consistency. Subjectively, it can minimize the overexposed and underexposed areas in the image under the premise of making full use of the camera’s dynamic range, so as to maximize the detailed information of the image. Objectively, although the average gradient of the image is relatively smaller compared with other methods, the number of saturated pixels in the image is the least and the signal-to-noise ratio is the highest, which exactly meets the quality requirements of remote sensing images.

**Figure 7:**
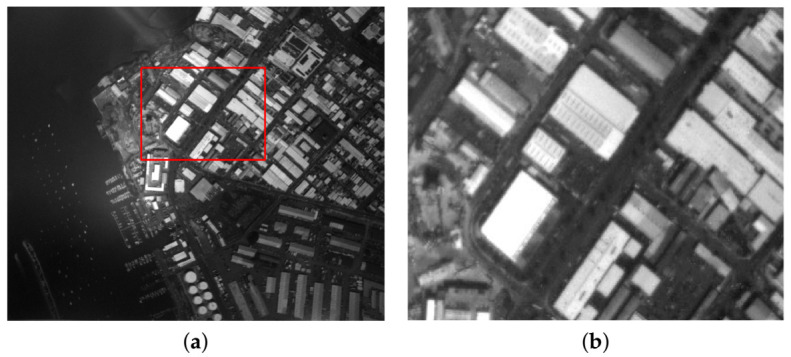
(**a**) Image taken with defocus; (**b**) enlarged view of the marked area.

During the experiment, it was found that other imaging parameters of the camera would have a certain effect on the accuracy of the algorithm, such as the focal length. In the series of experiments conducted, the camera was out-of-focus in one of the experiments. The optimal exposure image obtained is blurred, as shown in Figure 7. The overexposure area has not been effectively suppressed. After preliminary analysis, the image is blurred due to the defocus of the camera, which in turn causes the inaccuracy of the two-dimensional entropy matrix of the image and affects the segmentation of ROS and ROUS. Therefore, there is an error in the fitted exposure quality curve which ultimately affects the determination of the optimal exposure time. Research work would be carried out on the problem of accurate research of optimal exposure time for strong noise imaging systems.

## 4. Conclusions

In summary, a new automatic exposure method of plane array remote sensing image is proposed in this paper to appropriately evaluate the exposure quality of the image and then determine the optimal exposure time more accurately. Experiments conducted have proven that the theoretical model herein has certain advantages for image characteristics in terms of brightness, edges, information volume, and signal-to-noise ratio. It is more suitable for remote sensing imaging than current methods nowadays. The automatic exposure method is suitable for plane array space cameras, and it can also be rolled out to other types of cameras and applied in other engineering fields to solve the misuse of the camera’s dynamic range to obtain high-quality original images.

## Figures and Tables

**Table 1:** Technical parameters of acA1300-60gm camera.

Model	acA1300-60gm
Resolution (H×VPixels)	1280×1024
Chip access mode	CMOS global and rolling shutter
Pixel size (m)	5.3×5.3
Frame rate (fps)	60
Panchromatic/Bayer	Panchromatic

**Table 2:** Comparison of quantitative indices of the optimal exposure image of each method under bright conditions.

Index	CDM	AGLM	ODEM	EWGM	GBM	Proposed Method
*S*	0.0018	0.0012	0.0021	0.0012	0.0021	0.00021439
Average gradient	7.4980	7.1278	7.6342	7.1278	7.6342	5.8384
Ratio of saturated pixel/%	0.65	0.41	0.74	0.41	0.74	0.010529
SNR/dB	12.8128	13.5534	12.7366	13.5534	12.7366	15.9565

**Table 3:** Comparison of quantitative indices of the optimal exposure image of each method under dark conditions.

Index	CDM	AGLM	ODEM	EWGM	GBM	Proposed Method
*S*	0.0084	0.0108	0.00070877	0.0051	0.00070877	0.00001373
Average gradient	12.1817	12.7600	10.1319	11.2274	10.1319	8.3012
Ratio of saturated pixel/%	4.59	5.61	1.25	3.56	1.25	0.0047302
SNR/dB	11.2633	11.0264	12.4882	11.5696	12.4882	14.1612

## Data Availability

All data will be made available on request to the correspondent author’s email with appropriate justification.
